# Influence of hypobaric hypoxic conditions on ocular structure and biological function at high attitudes: a narrative review

**DOI:** 10.3389/fnins.2023.1149664

**Published:** 2023-05-09

**Authors:** Yuchen Wang, Xinli Yu, Ziyuan Liu, Zhongsheng Lv, Huaqin Xia, Yiren Wang, Jiaxi Li, Xuemin Li

**Affiliations:** ^1^Department of Ophthalmology, Peking University Third Hospital, Beijing, China; ^2^Beijing Key Laboratory of Restoration of Damaged Ocular Nerve, Peking University Third Hospital, Beijing, China; ^3^School of Biological Science and Medical Engineering, Beihang University, Beijing, China

**Keywords:** hypobaric hypoxia, ocular structure, biological function, high attitude, dynamic visual performance

## Abstract

**Background:**

With the development of science and technology, high-altitude environments, involving aviation, aerospace, and mountainous regions, have become the main areas for human exploration, while such complex environments can lead to rapid decreases in air and oxygen pressure. Although modern aircrafts have pressurized cabins and support equipment that allow passengers and crew to breathe normally, flight crew still face repeated exposure to hypobaric and hypoxic conditions. The eye is a sensory organ of the visual system that responds to light and oxygen plays a key role in the maintenance of normal visual function. Acute hypoxia changes ocular structure and function, such as the blood flow rate, and can cause retinal ischemia.

**Methods:**

We reviewed researches, and summarized them briefly in a review.

**Results:**

The acute hypobaric hypoxia affects corneal, anterior chamber angle and depth, pupils, crystal lens, vitreous body, and retina in structure; moreover, the acute hypoxia does obvious effect on visual function; for example, vision, intraocular pressure, oculometric features and dynamic visual performance, visual field, contrast sensitivity, and color perception.

**Conclusion:**

We summarized the changes in the physiological structure and function of the eye in hypoxic conditions and to provide a biological basis for the response of the human eye at high-altitude.

## 1. Introduction

With the development of science and technology, high-altitude environments, involving aviation, aerospace, and mountainous regions, have become the main areas for human exploration. However, such complex environments can lead to rapid decreases in air and oxygen pressure, for example, when the flight height changes significantly. Although modern aircrafts have pressurized cabins and support equipment that allow passengers and crew to breathe normally, flight crew still face repeated exposure to hypobaric and hypoxic conditions. Moreover, helicopters do not normally carry oxygen supply equipment. Thus, hypobarism and hypoxia can become an issue when flying over the plateau.

Human beings struggle to adapt to the hypobaric hypoxia caused by the high-altitude environment, and low-oxygen saturation can be harmful (Burtscher et al., 2021). For example, chronic hypobaric hypoxia can cause brain or lung edema, dyspnea, and emotional and consciousness disorders, while acute hypobaric hypoxia can lead to shock, myocardial infarction, and other adverse events (Petrassi et al., 2012).

The eye is a sensory organ of the visual system that responds to light and oxygen plays a key role in the maintenance of normal visual function. Acute hypobaric hypoxia changes ocular structure and function, such as the blood flow rate, and can cause retinal ischemia (Kaur et al., 2009), while chronic hypobaric hypoxia causes retinal neovascularization. Hypobaric hypoxia also has an impact on human color recognition, dark vision, and contrast sensitivity.

Flying an aircraft has a high visual demand and not only requires precise and timely visual acquisition, but also requires the ability to make accurate judgments based on the surrounding environment. During hypobaric hypoxia, these systems may be compromised, thus affecting the ability of pilots to perform tasks in civil and military situations and posing a potential threat to safety.

**Figure 1:**
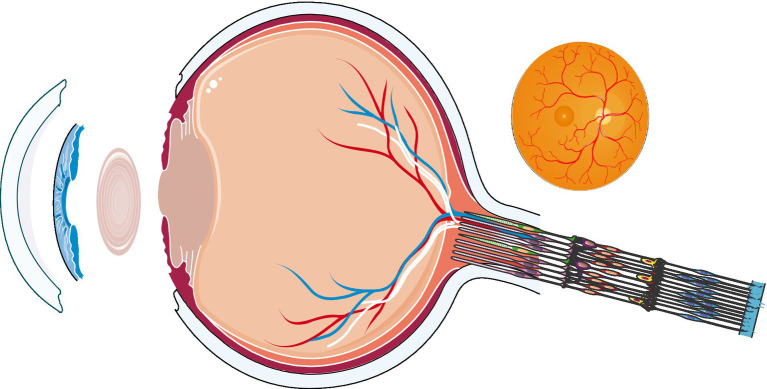
The overall structure of the ocular.

Therefore, the purpose of this literature review is to clarify the changes in the physiological structure and function of the eye in hypoxic conditions and to provide a biological basis for the response of the human eye at high-altitude (Figure 1).

## 2. The influence of hypobaric hypoxia on ocular structure

### 2.1. Influence on the cornea

Studies have shown that the eyes are one of the most hypoxia-sensitive organs (Akberova et al., 2016). High-altitude exposure affects the normal function of the optical pathway, especially the cornea (Nebbioso et al., 2014), and causes structural changes at different levels of hypobaric hypoxia. Willmann et al. (2013) studied the changes of ocular surface caused by chronic hypobaric hypoxia at high altitudes in 14 healthy adults, who ascended from 1,635 m to 4,559 m within 6 h on a mountain, and stayed at 4,559 m for 4 days. On the day 1 of rapid elevation, the thickness of tear film and the corneal epithelium significantly decreased, while the thickness of the corneal endothelium and corneal stroma significantly increased. Corneal thickness continued to increase until day 4, but rapidly decreased to the baseline level when the group returned to sea level. Thus, staying at 4,559 m for 4 days induced moderate chronic hypobaric hypoxia. Chronic hypobaric hypoxia causes anaerobic glycolysis in corneal epithelial cells and lactic acid accumulation. Eventually, the lactic acid diffuses through the corneal stroma and endothelium and metabolizes in the aqueous humor, leading to osmotic pressure-dependent aqueous humor reflux. This reduces corneal endothelial pump activity and leads to corneal edema and increased corneal thickness (O'Leary et al., 1981). In the lack of oxygen, corneal edema happens, claim Pang et al. (2021). This finding further indicates that corneal edema caused by ATP deficiency and pH changes as a result of lactate/CO_2_ accumulation and increased glycolysis are the causes of hypoxia-induced edema.

Akberova et al. (2016) identified the effects of acute hypobaric hypoxia on conjunctival and corneal epithelium. *In vitro* experiments, whereby mice were placed in an acute hypoxic environment with a pressure of 180 mmHg for 3 min, revealed intracellular DNA breakage and apoptosis in conjunctival and corneal epithelial cells, but not corneal stromal cells. Therefore, it was proposed that conjunctival and corneal epithelial cells initially respond to environmental changes, and activate a protective mechanism to prevent the corneal stroma from being affected by substance metabolism during acute hypobaric hypoxia. As a result, when the cells undergo apoptosis, the normal tear film is damaged, the ocular surface environmental homeostasis is disrupted, and ocular surface diseases such as dry eyes occur.

Therefore, regardless of acute or chronic hypobaric hypoxia, we can predict that high altitudes lead to a disorder in corneal collagen arrangement, a decrease in the stability of the corneal epithelium and tear film layer, an increase in functional irregularity, and the occurrence of clinical symptoms such as dry eye symptoms, decreased vision, and astigmatism. These symptoms can subsequently affect the judgment of flight personnel using the complex instrument panels on aircrafts during flights at high-altitude, posing a threat safety.

### 2.2. Influence on anterior chamber angle and depth

Acute or chronic angle-closure glaucoma can occur following drastic changes in chamber angle and depth, resulting in vision loss, visual field defects, and serious harm to the optic nerves. Hence, it is important to study the influence of hypobaric hypoxia on the anterior chamber angle (ACA) and anterior opening distance (AOD). Interestingly, in the previous study, the AOD and ACA decreased during the elevation from 1,635 to 4,559 m, and showed a downward trend during the stay at 4,559 m. However, no significant changes in AOD or ACA during exposure to high altitude were noted (Willmann et al., 2013). Similarly, Jatinder et al. demonstrated that there was no statistical difference in AOD long-term follow-up data between residents living at an altitude of 3,300 m and residents living at an altitude of 1,700 m (Bali et al., 2005). Thus, long- and short-term stays in a high-altitude environment may not affect the AOD.

### 2.3. Influence on pupils

The pupil is an indicator that reflects the effect of hypobaric hypoxia on the central nervous system (CNS). Cymerman et al. (2005) investigated oculomotor reflexes, including pupil diameter (PD), constriction amplitude (CA), constriction latency (CL), and saccadic velocity (SV) during acute hypobaric hypoxia over a period of 2 weeks at 4,300 m with measurements taken every 2 days. They found that PD, CA, and CL were significantly lower than baseline levels and remained up to 14 days (Krusche et al., 2020). Additionally, under different light intensities of 12 cd/m^2^, 1 cd/m^2^, and 0.1 cd/m^2^, the corresponding PDs were 0.55 mm, 0.46 mm, and 0.29 mm, respectively. These values then decreased by 14, 11, and 8% when exposed to hypobaric hypoxia and increased by 9, 5, and 1% under normal oxygen concentration conditions. Thus, exposure to high altitude causes hypoxic myosis, which promotes pupil sphincter activity in the Edinger–Westphal nucleus of the oculomotor nerve in the mesencephalon, leading to pupil constriction. The retinal afferent light stimulation and supranuclear inhibitory pathways are crucial factors in regulating pupil size. When light is reduced, the supranuclear inhibitory pathway is relatively activated, and hypobaric hypoxia inhibits the activation of the central and parasympathetic nervous systems; thus, constricting the pupil.

### 2.4. Influence on crystal lens

The influence of acute or chronic hypobaric hypoxia on the lens has not yet been studied in the human eye. The lens naturally exists in a hypoxic environment due to the lack of blood supply, resulting in a decrease in the oxygen concentration from the lens surface to the core. According to a study by Shui et al. (2006), oxygen is distributed differently in various rabbit eye tissues. Under hyperoxic conditions (21% O_2_), oxygen concentration was the lowest at the posterior surface of the lens, and decreased more obviously as oxygen levels decreased. Hypobaric hypoxia did not cause changes in the lens structure. Shui and Beebe (2008) subsequently measured the proliferation rate of lens epithelial cells under high- and low-oxygen concentrations *in vitro* and found that the proliferative activity of the cells did not increase when the oxygen level was below normal. In contrast, hypobaric hypoxia has been shown to cause lens maturation through the activation of hypoxia-inducing factor (HIF1a) (Brennan et al., 2020), which regulates hypoxia-responsive genes and promotes the elimination of organelles in epithelial cells, enabling them to differentiate into lens fibrocytes and achieve regular arrangement. In addition, hyperoxia is associated with age-related cataracts. Zhang et al. (2010) studied the rat lens and revealed that oxygen depletion did not induce reactive oxygen species (ROS). Moreover, there was no effect on the gene expression of mitochondrial DNA (mtDNA) and mtDNA base excision repair enzyme (mtBER) in the lens following hypobaric hypoxia, and the nucleus remained transparent. However, ROS were induced when the oxygen concentration increased to 60%, and following a significant increase in oxygen consumption in the lens, the lens fibers became irregular. Consequently, normal age-related lens growth required relatively low-oxygen levels. Overall, hypobaric hypoxia has no effect on the lens.

### 2.5. Influence on vitreous body

The retina has two major systems that supply oxygen. The outer plexus, where photoreceptor cells are located, receives nutrients through choroid blood circulation, while the inner retina is mainly supplied by the shallow and deep capillary plexuses of the central retinal artery branch. As such, the inner retina is more sensitive to hypobaric hypoxia. Under hypoxic conditions, a large number of soluble factors such as cytokines, chemokines, and growth factors are secreted into the vitreous cavity (dell'Omo et al., 2013). Cytokines are involved in cell proliferation, inflammation, immunity, tissue repair, and other biological processes. They can enhance immune responses by directing the recruitment of leukocytes to sites of inflammation. Growth factors are involved in diabetic retinopathy. During hypobaric hypoxia, macrophages collect in low-oxygen areas, express monocyte chemoattractant protein-1, and release tumor necrosis factor α (TNF-α), which causes the release of interleukin 8 (IL-8) and vascular endothelial growth factor (VEGF) and eventually results in retinal vasculopathy. Hence, although there is currently no relevant study on the vitreous in acute or chronic hypobaric hypoxia, we cannot underestimate the effect of hypobaric hypoxia on the vitreous body. Further studies are needed to determine whether hypobaric hypoxia affects vitreous composition, liquefaction, and early post-detachment.

### 2.6. Influence on retina

The retina is a highly differentiated neural structure with poor hypobaric hypoxia tolerance, which causes retinal artery spasm, blood stasis, and an increase in venous pressure and tortuous filling. These can then lead to retinal hemorrhage, macular edema, and even acute retinal artery occlusion following extreme or transient vision loss. Therefore, it is very important for pilots to understand the influence of hypobaric hypoxia on the retina when they encounter emergencies at high altitudes.

Kaur et al. (2009) studied a rat model exposed to 5% oxygen and 95% nitrogen for 2 h and found that hypobaric hypoxia led to damage of the retinal cell structure, increased VEGF concentration and NO production, increased vessel permeability (such that Müller cells became swollen), and neural cell degeneration (Shinojima et al., 2021). Additionally, in rat models exposed to 10% oxygen for 48 h (Mesentier-Louro et al., 2020), hypobaric hypoxia increased expression of proapoptotic transcriptional regulator CCAAT-enhancer-binding protein homologous protein (CHOP) in glial cells in the retina and optic nerve. Histological, immunofluorescence, and morphometric analyses revealed a significant increase in CHOP immunoreactivity in astrocytes in all layers of retinal neurons and in intra-retinal, retro-bulbar, and anterior myelinated optic nerves. As CHOP is a marker of endoplasmic reticulum (ER) stress, we can conclude that hypobaric hypoxia leads to severe retinal stress injury. In addition, hypobaric hypoxia induced an obvious increase in glial fibrillary acidic protein (GFAP), which is associated with the reactive activity of astrocytes, especially in the retina and myelinated nerve fibers. Oligodendrocytes are particularly vulnerable to hypoxic ischemia and it was found that the number of oligodendrocytes obviously decreased during hypobaric hypoxia, indicating that glial function can be impeded in the early stages of hypobaric hypoxia.

In chronic hypobaric hypoxia, the metabolism and survival function of the pigment epithelium and photoreceptors in the outer layer are affected. A longitudinal study by Ebner et al. (2021) conducted over 11 weeks in hypobaric hypoxia at 3,450 m revealed a shortening of photoreceptor segment length, which may indicate that longer durations of hypobaric hypoxia exposure gradually decreases the color discrimination ability and sensitivity of the human eye. However, this has yet to be observed in human studies.

Hypobaric hypoxia can also cause capillary overperfusion (Neumann et al., 2016), resulting in vasogenic cerebral edema. The retina is the only part of the CNS that can observe and measure capillary blood flow. Therefore, it is very important to identify the regulatory properties of the retina and choroid during hypobaric hypoxia. Bosch et al. (2009) observed changes in retinal blood vessels in 27 individuals at different altitudes over 2 weeks and found that the diameter of retinal blood vessels increased significantly, especially arteries, at a height of 4,497 and 5,533 m, but returned to baseline levels when the altitude dropped. Therefore, high altitudes triggered retinal blood vessel dilation. Moreover, retinal blood flow velocity steadily increased as altitude increased, and peaked at 6,265 m. Afterward, the velocity decreased despite further ascent. This may be related to the vessel diameter, perfusion pressure, blood viscosity, or significantly increased hematocrit. Nevertheless, this showed that retinal blood is sensitive to changes in oxygen concentration and adapts quickly (Baertschi et al., 2016). In contrast, the choroidal capillary flow velocity around the macular fovea did not increase at 4,497 and 5,533 m, but did increase and remained stable after ascending to 6,265 m. The choroid oxygen transport capacity was also relatively high whereas oxygen levels were highly reserved, such that the choroidal blood flow extracted low oxygen, yet remained relatively stable. Frayser et al. (1974) also found that retinal blood flow increased by 89% after 2 weeks of high-altitude exposure at 7,456 m. Following an increase in the duration and altitude of exposure to the plateau environment, blood flow increased by 128% after 1 week and 174% after 7 weeks. Thus, the mechanism of adaption ensured oxygen delivery to the retina. However, there was a steady decline in retinal blood flow velocity after an initial increase, which was associated with increased blood viscosity and hematocrit. Moreover, a case of high-altitude retinopathy with vitreous hemorrhage in 4,760 m high, was reported by Shrestha et al. (2021), the vascular bed or capillaries of tiny arterial collateral leaks are what cause retinal hemorrhage. Due to the increased blood flow and flow velocity, these capillaries may become more brittle. Increased cerebral blood flow and raised cerebral venous pressure are the results of changes in the circulatory and respiratory physiology brought on by changes and abnormalities in retinal hemodynamics and hypobaric hypoxia. These impacts reduce the uptake of cerebrospinal fluid, which causes hypobaric hypoxia to increase cerebrospinal fluid and cause papilledema.

In summary, we found that hypobaric hypoxia led to increased retinal vascular permeability *in vitro*, VEGF and NO metabolite release, ER stress, and ultimately apoptosis and degradation of retinal glial cells and neurons. Hypoxia-induced vascular dilation and increased blood flow velocity have also been observed in humans. Schatz et al. (2013) assessed the functional integrity of retinal layers at 341 and 4,559 m using electroretinography (ERG). The data showed a change in retinal function in the inner, outer, and ganglion cell layers, with the cone-rod response (phototransduction and visual processing) being the most vulnerable, suggesting that cone and rod function may be affected by high-altitude exposure. Furthermore, hypobaric hypoxia may contribute to adverse events such as retinal blood vessel bleeding and loss of photoreceptor cell function.

As such above, hypoxia has a significant effect on the structure of all parts of the human eye, thus affecting visual function.

## 3. Influences on visual function

Clarifying the influence of acute and chronic hypobaric hypoxia on ocular structure is conducive to our study of the influence of hypobaric hypoxia on visual function.

### 3.1. Influence on vision

Vision is the most intuitive indicator of the effect of hypobaric hypoxia on the human eye. Gekeler et al. (2019) explored changes in vision in the plateau environment (4,559 m) after 3 days. The average best-corrected visual acuity (BCVA) of participants was −0.19 logMAR at ground level. The average BCVA was 0.01 logMAR on day 2 at 4,559 m, and 0.05 logMAR on day 3 of 4,559 m. A gradual decline in visual acuity was observed, but this was not statistically significant. Winkle et al. (1998) evaluated the effects of hypobaric hypoxia on participants who previously underwent radial keratotomy (RK) surgery, whereby participants were subjected to ocular surface hypobaric hypoxia for 2 h, and found a significant tendency of hyperopic shift and corneal flattening. However, in an experiment conducted by Nelson et al. (2001), the opposite result was found following exposure to hypoxic conditions on the ocular surface in 20 participants who previously underwent laser *in situ* keratomileusis (LASIK) surgery, who experienced obvious myopia drift occurred and corneal steepening. Until now, there has been no literature on specific changes in visual acuity following hypobaric hypoxia exposure; thus, further verification is needed.

### 3.2. Influence on intraocular pressure

The high-altitude hypobaric hypoxia environment clearly effects intraocular pressure (IOP) (Albis-Donado et al., 2018; Najmanova et al., 2018). Yang et al. (2019) and Bruttini et al. (2020) illustrated that within the moderate altitude range of 1,300 m (19°C) to 3,466 m (−1.4°C), the average IOP at 3,466 m was statistically lower than that at sea level, and altitude significantly correlated with IOP. Subsequently, Willmann et al. (2017) studied IOP at 4,559 m after 3 days and found no significant change compared with baseline levels. However, IOP after corneal thickness correction was measured and was significantly lower than baseline.

Conversely, Wu et al. (2020) measured the IOP of 20 participants after 7 days at 3,658 m (Beijing to Tibet) and found the mean IOP was statistically higher than baseline. In addition, Najmanova et al. (2018) studied IOP at 6,200 m for 4 and 10 min and found that the mean IOP increased by 1.2 mmHg and 0.9 mmHg, respectively, but returned to the baseline level when oxygen was restored. This indicates an upward trend in IOP as altitude increases.

Overall, the studies relating altitude and IOP were mixed; therefore, it was necessary to summarize the relevant results. Yang et al. (2019) conducted a meta-analysis on IOP changes at different altitudes. The data showed that IOP significantly decreased with the increase in altitude between 3,000 and 5,500 m, whereas IOP increased at extreme altitudes of over 5,000 m. They also found that a duration of exposure of more than 72 h was likely to induce a decrease in IOP. A potential reason for this is that the decrease in blood oxygen saturation (Yang and Wang, 2022) at higher altitude inhibits carbonic anhydrase activity and affects the formation of aqueous humor. Alternatively, inhibition of pigmentation-free epithelial cells of the ciliary body may result in decreased aqueous humor production and IOP. The temperature in the plateau environment was significantly lower than the baseline level, causing the local microarteries contracted after exposure and resulting in a decrease in superficial scleral vein pressure; thus, reducing the outflow resistance of the aqueous humor and IOP. Moreover, corneal metabolism switches to anaerobic metabolism in an anoxic environment. This leads to the accumulation of extracellular metabolites and increased extracellular osmotic pressure, which causes corneal edema and increases central corneal thickness and IOP. In summary, many factors affect IOP, but these variations are not obvious. Therefore, we hypothesize that the high-altitude environment has little influence on IOP, is relatively safe, but needs to be further clarified.

### 3.3. Influence on oculometric features and dynamic visual performance

During high-altitude flight, pilots need to obtain timely information from the display control interface under motion; therefore, it is important to identify the effect of hypobaric hypoxia on dynamic vision and oculometric features. Data related to ocular movement are direct indicators of biological cognitive activity. Stepanek et al. (2014) assessed oculometric features such as blink metrics, PD, fixations, and saccades under different hypoxic conditions [hypoxic hypoxia (HH) with 8% O_2_ and isocapnic hypoxia (IH) with 7% O_2_ + 5% CO_2_ + balance N_2_]. They found that in HH and IH, the blink rate increased by a factor of 1 compared to the baseline level, and the blink rate was faster in HH than in IH. In addition, the blink interval and duration decreased with an increase in blink frequency. Faster recovery of the blink rate occurred when transitioning to normoxia. As for pupil movement, the proportion of PD that significantly increased under HH conditions was significantly higher than that which occurred under IH conditions. PD returned to baseline levels when the oxygen content returned to normal. This may be partly because hypobaric hypoxia activates the sympathetic nervous system, leading to increased dopamine release and blink rate (Panjwani et al., 2006).

Additionally, the time of pupil fixation increased by 8% under HH and 0.4% under IH, but pupil saccade function, such as average saccade length, total saccade times, saccade amplitude, and saccade velocity were not significantly different under different hypoxic conditions. Ocular fixation and micro-saccades are considered as indicators of attention and cognition. Micro-saccades can help counteract visual adaptation by shifting retinal images during movement to maintain visibility during fixation. We found that increased fixation time is required in anoxic environments, suggesting that hypobaric hypoxia may lead to impaired fixation stability. Therefore, coulometric features and access to information may be affected when hypobaric hypoxia occurs during flight.

As dynamic visual performance is an important factor in ensuring the safety of high-altitude flight, Krusche et al. (2020) studied the changes in dynamic visual performance of healthy individuals at 3,647 and 4,554 m. Under the condition that the brightness of the display and the distance from human eyes were stable and consistent, they detected four different motion contrasts: 100, 50, 30, and 20%. The results showed that for 30 and 20% contrast, the dynamic vision performance gradually decreased as the altitude increased, while under 20% contrast, the dynamic vision above the fovea of the macula decreased significantly. Therefore, we believe that hypobaric hypoxia affects the dynamic vision and increases the risk of in-flight accidents.

### 3.4. Influence on visual field

Through our literature review, we learned that hypobaric hypoxia effects eye movement and dynamic vision. According to the study of Krusche et al. (2020), dynamic vision above the macula fovea significantly changes during hypobaric hypoxia, which suggests that the visual field may also change. Therefore, it is necessary to review the effects of hypobaric hypoxia on the visual field. Horng et al. (2008) studied 15 healthy young male pilots with a mean arterial oxygen saturation (SaO2) of 99% and a mean visual field sensitivity of 43.9 dB at ground level. When the altitude increased to 7,620 m, SaO_2_ dropped to 64% within 3 min and the mean visual sensitivity was significantly reduced by 7.2 dB. In the range of 0°–10°, the visual sensitivity decreased by 6.1 dB on average. In the range of 10°–20°, the visual sensitivity decreased by 7.0 dB. In the range of 20°–30°, the peripheral visual sensitivity decreased by 8.3 dB. Therefore, peripheral visual sensitivity diminished more than central sensitivity. Furthermore, Feigl et al. (2011) analyzed absolute sensitivities (in dB) at 1°, 3°, 6°, 10°, 15°, 22°, and 30° eccentricities, and the mean defect (MD) and pattern defect (PD) were calculated by static and flicker visual perimetry. Under photopic illumination, flicker and static visual field sensitivities at all eccentricities, or MD and PD, were not significantly different between hypoxic and normoxic conditions. However, the static field was more sensitive than the flicker field in detecting low-sensitivity areas under hypoxic and normal oxygen conditions.

These findings are consistent with previous studies that have shown that the retina is unable to perform its normal physiological functions under hypobaric hypoxia, ER stress, or photoreceptor cell damage.

The decrease in the peripheral visual field was more obvious than that in the central visual field during hypobaric hypoxia. This may be related to the accumulation of rods in the peripheral visual field and distribution of cones in the central part of the retina. Hypobaric hypoxia leads to an increased threshold of rods and cones in the visual field, and a decreased response to light stimulation. Thus, visual field sensitivity decreased with a decrease in oxygen concentration.

### 3.5. Influence on contrast sensitivity

Contrast sensitivity (CS) is a very important function for pilots to observe their surroundings during night flights. Connolly and Hosking (2009) preliminarily explored the CS threshold under hypoxic conditions at 3,048 m and found that the sensitivity threshold significantly correlated with oxygen at ~1 cd/m^2^ and that contrast acuity obviously decreased and increased after 100% oxygen inhalation. Therefore, oxygen supply can enhance dynamic CS during flight, which is conducive to flight safety.

Gekeler et al. (2019) also investigated the change in CS during hypobaric hypoxia. The average CS at ground level was 1.28 logCS, while the average CS at 4,559 m was 1.03 logCS on day 1, −0.10 logCS on day 2, and −0.12 logCS on day 3. Therefore, the CS significantly decreased at 4,559 m, which positively correlated with oxygen saturation.

Hypobaric hypoxia also affects night vision, which includes light sensitivity in the peripheral and central parts of the retina. Gekeler et al. (2019) confirmed that night vision gradually decreased under hypobaric hypoxia conditions as altitude increased, and that the dark adaptation threshold increased and delayed the dark adaptation peak. Under mesopic conditions at dusk, the oxygen consumption of photoreceptors, especially rods, is enhanced compared to bright light conditions, making the outer retina more sensitive to low oxygen at light levels associated with night flight. Connolly and Barbur (2009) examined the oxygenation state on the contrast thresholds required to maintain visual acuity at low photopic (12 cd/m^2^), upper mesopic (1 cd/m^2^), and mid-mesopic (0.1 cd/m^2^) luminance. They found that the contrast threshold increased at all light levels, particularly at 1 cd/m^2^ to 0.1 cd/m^2^, relative to normoxia. Hypobaric hypoxia increases contrast thresholds; thus, visual performance between ~10 cd/m^2^ and 0.1 cd/m^2^ is oxygen-dependent.

Under dim conditions, contrast sensitivity decreases due to low photopic vision, glare, and shadows. However, hypobaric hypoxia has a more significant effect on contrast sensitivity. According to a previous study, hypobaric hypoxia led to dysfunction of photoreceptor cells under dim conditions. Contrast sensitivity may be further decreased as dark adaptation is prolonged. Consequently, changes in pupil size under dim light may also affect the appearance of ocular problems, such as high-order aberrations. Thus, as the influence of the pupil was not excluded in the above experiments, this should be clarified further.

### 3.6. Influence on color perception

Connolly and Barbur (2009) showed that hypobaric hypoxia impacted the ratio of red to green in the color scope test in individuals with normal color vision (NCV), such that the ratio of green fluorescence in NCV individuals was lower than that under normal oxygen concentrations.

Hovis et al. (2012) revealed that color assessment and diagnosis (CAD) test results at 3,780 m above sea level suggested that the red-green thresholds of NCV patients relatively increased, while the yellow-blue thresholds did not notably change. Conversely, Connolly et al. (2008) found that hypobaric hypoxia significantly impaired color sensitivity at the lowest light level, in which red-green and yellow-blue thresholds were noticeably impaired, with the latter most affected. Yellow-blue threshold asymmetry obvious at the lowest light level, such that the yellow threshold corresponded to an increase in the short-wave sensitive cone signal, which was more damaged than the complementary blue threshold, indicating that the increase and decrease in the short-wave sensitive cone signal were asymmetrical at low light levels.

Connolly and Serle (2014) investigated the ability to recognize information using night-vision equipment and dim dashboards under hypoxic conditions, and noted the changes in recognition thresholds after inhaling oxygen. They hypothesized that mild breathing disorders may lead to a decrease in color sensitivity. When the background color of the night-vision instrument was green, the brightness was 1.0 cd/m^2^ and 3.0 cd/m^2^, while the dim instrument panel was 1.0 cd/m^2^ and 0.1 cd/m^2^. Color threshold discrimination was detected under normal (air), hyperoxic (100% O2), and hypoxic (13.7% O2) environments. They found that oxygen was an important factor when discriminating color, as the red-green and rod-related color thresholds were enhanced by 20–25% during hypobaric hypoxia and enhanced by 50% when using the night-vision device. Hypobaric hypoxia leads to increased metabolic demand and slowed signal conduction; hence, hypobaric hypoxia at high altitudes has a certain impact on the recognition ability of human color vision.

Hovis et al. (2012) revealed the relationship between altitude and color sensitivity in individuals with NCV and red-green vision deficits. The color threshold was measured using the Cambridge color test (CCT), the color assessment and diagnosis (CAD) test, and the cone specific contrast test (CSCT) at the ground and at 3,780 m. CAD showed that the red-green threshold slightly increased by 10% for trichromatic individuals, while the yellow-blue threshold did not significantly change. For dichroic individuals (i.e., the red-green-deficient population), the blue-yellow threshold slightly increased. Meanwhile, the red-green threshold did not notably increase and no significant changes were observed in the other colors. CCT and CSCT did not reveal any significant changes in chromatic thresholds. The oxygen concentration at which color perception begins to be impaired corresponds to ~2,400 m above sea level. As the altitude increased to 3,000 m, the range of impaired color discrimination began to involve visible light, and the loss of color discrimination became more pronounced above 4,000 m. In conclusion (Temme et al., 2017), the color sensitivity of human eyes at low light levels changes, especially the asymmetric change in the yellow-blue threshold during hypobaric hypoxia. Rod density increases and cone density decreases further away from the fovea, and the color sensitivity is more susceptible to the influence of hypobaric hypoxia. However, the mechanism remains unclear (Table 1).

**Table 1:** Effects of hypoxia on ocular structure and function.

Subjects	Age (number)	Hypoxia conditions	Main results	Citation	Duration of hypoxia
Rats	3 months (20)	Hypoxia was created by pumping air from the pressure chamber for 1 min before reaching a pressure of 180 mm Hg	The DNA were damaged and apoptosis in the anterior epithelium of the cornea and conjunctiva	Akberova et al. (2016)	1 min
Rats	1-day-old	Hypoxia was created by a chamber filled with a gas mixture of 5% oxygen/95% nitrogen	Muller cell processes were swollen in the inner nuclear layer, ganglion cells were swollen and mitochondria were disrupted	Kaur et al. (2009)	2 h
Rats	6–8 weeks	Hypoxia was created by a chamber filled with 20.9–10% oxygen	Hypoxia induced thinning of the retina on OCT but little cell loss on histology. Hypoxia led to significant reduction of total number of oligodendroglia in the optic nerve	Mesentier-Louro et al. (2020)	48 h
Rats	9 weeks (6)	Mice were exposed to short-term hypobaric hypoxia at 3,450 m above sea level	Rod segment length showed a significant shortening	Ebner et al. (2021)	48 h
Healthy huamns	Not specific (6)	5,334 m	The circulated retinal blood flow of 367 mL·100 g^−1^·min^−1^ was significantly greater than the flow of 331 mL·100 g^−1^·min^−1^	Frayser et al. (1974)	5 days
Radial keratotomy (RK) huamns	26–58 years (20)	Humidified compressed air (20% oxygen) was filtered on one side and humidified 100% nitrogen (0% oxygen) on the other side. (sealed goggle-type microenvironment chamber)	A significant hyperopic shift and corneal flattening occurred in all subjects with RK compared with those of control subjects	Winkle et al. (1998)	2 h
Healthy humans	26–58 years (20)	Humidified compressed air (20% oxygen) was filtered on one side and humidified 100% nitrogen (0% oxygen) on the other side. (sealed goggle-type microenvironment chamber)	A significant increase in corneal thickening occurred	Winkle et al. (1998)	2 h
Laser *in situ* keratomileusis (LASIK) humans	38.8 years (20)	Humidified nitrogen (airtight goggle system)	A significant myopic shift occurred in LASIK corneas exposed to hypoxia compared with myopic control subjects. A significant increase in corneal thickening occurred	Nelson et al. (2001)	2 h
Healthy humans	38.8 years (20)	Humidified nitrogen (airtight goggle system)	A significant increase in corneal thickening occurred	Nelson et al. (2001)	2 h
Healthy humans	Not specific	4,300 m (transport)	The pupil diameter and constriction latency decreased. The constriction amplitude decreased. The saccadic velocity increased	Cymerman et al. (2005)	48 h
Healthy humans	31.4 (15)	7,620 m (simulated)	Mean visual sensitivity was significantly reduced. Peripheral sensitivity was slightly but significantly more diminished than central sensitivity	Horng et al. (2008)	3 min
Healthy humans	30	3,048 m (mask)	At mesopic luminance (1 cd/m^2^), sensitivity was consistently poorest with hypoxia and greatest with supplementary oxygen at all eccentricities and in all field quadrants, suggesting oxygen-dependent performance. Hypoxia significantly impaired color sensitivity at the lowest light level, in which red-green and yellow-blue thresholds were noticeably impaired	Connolly and Barbur (2009)	1 h
Healthy humans	43 years (27)	4,497 m (hike) 5,533 m (hike) 6,265 m (hike) 7,546 m (hike)	The initial increase in macular retinal blood velocity was followed by a decrease at higher altitudes despite further ascent, whereas choroidal flow increase occurred later, at even higher altitudes	Bosch et al. (2009)	4,497 m–3 d 5,533 m–3 d 6,265 m–6 d 7,546 m–3 d
Healthy humans	20.9 ± 0.5 (14)	Breathing 12% oxygen (hypoxia)	Under photopic illumination, flicker and static visual field sensitivities at all eccentricities were not significantly different between hypoxia and normoxia conditions	Feigl et al. (2011)	3 min
Healthy humans	37.6 ± 12.6 (13)	3,780 m (stimulated)	The red-green thresholds of NCV patients relatively increased, while the yellow-blue thresholds did not notably change	Hovis et al. (2012a)	4.5 h
Healthy humans	25–54 years (13)	4,559 m (cable car)	The maximum response of the scotopic sensitivity function, the implicit times of the a- and b-wave of the combined rod-cone responses, and the implicit times of the photopic negative responses (PhNR) were significantly altered. The most affected ERG parameters are related to combined rod-cone responses, which indicate that phototransduction and visual processing, especially under conditions of rod-cone interaction, are primarily affected at high altitude	Schatz et al. (2013)	48 h
Healthy humans	36 ± 9 years (14)	4,559 m (trekking)	A significant increase of CCT during altitude exposure due to stromal edema were shown. This change was completely reversible upon descent	Willmann et al. (2013)	48 h
Healthy humans	32 ± 5 (20)	5,486 m (hypobaric chamber with 10% oxygen mask)	Pachymetry values related to corneal thickness in conditions of hypobarism revealed a statistically significant increase	Nebbioso et al. (2014)	Not specific
Healthy humans	32.4 ± 9.8 (25)	7,894 m (mask)	Blink rates were significantly increased under hypoxic conditions. The percentage change in pupil size fluctuation was increased. Total saccadic times under hypoxic conditions were significantly increased compared with normoxia	Stepanek et al. (2014)	3 min
Healthy humans	32.8 ± 5.8 (12)	Breathing 13.7% oxygen, balance nitrogen	Contrast acuity thresholds were elevated consistently under hypoxia by up to 25% relative to breathing oxygen	Connolly and Serle (2014)	1 h
Healthy humans	46.6 ± 7.8 years (33)	6,000 m (trekking)	Retinal venous pressure and ocular perfusion pressure changed significantly at both high altitude of 4,200 and 6,000 m	Baertschi et al. (2016)	Not specified
Healthy humans	36.7 ± 10.8 (17)	3,000 m (funicular)	Retinal arterial and venous diameter increased, arterial and venous response to flicker light decreased	Neumann et al. (2016)	48 h
Healthy humans	34.9 (26)	Breathing 12% oxygen 87% nitrogen (diluting tanked)	Under mesopic hypoxia conditions, the known high-oxygen demand of rods may reduce the retinal oxygen available for cones thereby diminishing color sensitivity as well as other cone functions	Temme et al. (2017)	15 min
Healthy humans	41.7 ± 9.4 (41)	2,234 m (sea level)	Mean IOP with Dynamic contour tonometry showed not a significant difference. Mean Goldmann applanation tonometry IOP at the two altitudes was statistically significant difference	Albis-Donado et al. (2018)	24 h
Healthy humans	25.2 ± 3.8 (38)	6,200 m (simulated)	The hypoxia induced changes in intraocular pressure were significantly correlated with the arterial oxygen saturation changes, whereas the relationship with intraocular pressure baseline and initial heart rate were insignificant	Najmanova et al. (2018)	10 min
Healthy humans	35 ± 8 years (14)	4,559 m (cable car)	A significant decrease in contrast sensitivity (CS) was found for Weber CS at high altitude compared with baseline. Visual acuity remained unchanged	Gekeler et al. (2019)	48 h
Healthy humans	24 ± 2.5 year (11)	4,554 m (hike)	A significant reduction in dynamic visual performance in the superior parafoveal retinal subfield, partly representing the lower visual field	Krusche et al. (2020)	48 h
Healthy humans	24.0 ± 2.5 (11)	4,223 m (hike)	A significant reduction in dynamic visual performance in the superior parafoveal retinal subfield, partly representing the lower visual field	Krusche et al. (2020)	6 days

## 4. Discussion

In summary, visual perception activities in high-altitude flights are more complex and involves not only static vision but also dynamic vision, which encompasses the observer’s ability to recognize dynamic target details. High-altitude hypoxic environments cause changes in the physiological structure of the eye, such as corneal thickness and rod and cone cell density, and in biological optics, such as diopter state changes. These changes affect the overall visual function of the human eye, such as color discrimination, color sensitivity, the resolution of external objects under different light intensities, such as contrast sensitivity, and the recognition of moving objects from different aspects. However, much is still unknown, especially with regard to color sensitivity as changes in red-green and yellow-blue perception thresholds can lead to serious visual difficulties for pilots when flying at the plateau. Therefore, further research on the changes in visual color perception caused by the plateau environment is required.

## Author contributions

YuW reviewed the literatures and drafted the manuscript. XY, HX, JL, ZhL, and YiW participated in the translation of articles. ZiL reviewed the manuscript. XL gave final approval of the version to be submitted and any revised version. All authors contributed to the article and approved the submitted version.

## Funding

This study was supported by Beijing Natural Science Foundation, grant number 7202229.

## Conflict of interest

The authors declare that the research was conducted in the absence of any commercial or financial relationships that could be construed as a potential conflict of interest.

## Publisher’s note

All claims expressed in this article are solely those of the authors and do not necessarily represent those of their affiliated organizations, or those of the publisher, the editors and the reviewers. Any product that may be evaluated in this article, or claim that may be made by its manufacturer, is not guaranteed or endorsed by the publisher.
